# Nervous Disturbances of Pregnancy Due to Defective Excretion1Read before the Buffalo Medical Club

**Published:** 1893-05

**Authors:** C. C. Frederick

**Affiliations:** Buffalo, N. Y., Adjunct Professor of Obstetrics, Medical Department of Niagara University; Obstetrician and Gynecologist to the Buffalo Woman’s Hospital; Obstetrician to the St Mary’s Widows’ and Infants’ Asylum; 64 Richmond Avenue


### NERVOUS DISTURBANCES OF PREGNANCY DUE TO DEFECTIVE EXCRETION.[1]

By C. C. FREDERICK, M. D., Buffalo, N. Y.,

Adjunct Professor of Obstetrics, Medical Department of Niagara University; Obstetrician and Gynecologist to the Buffalo Woman's Hospital; Obstetrician to the St Mary's Widows' and Infants' Asylum

The mere existence of pregnancy is too often considered as the cause of the nervous disturbances associated with it. An unimpregnated woman may suffer from functional nervous disorders and have the causes correctly ascertained. Why a pregnant woman should not be accorded the same consideration, I know not. The text-books on obstetrics usually devote a space to the nervous disorders of pregnancy. This, naturally, presupposes that because a woman is pregnant she will have some neurotic troubles. It must be admitted that pregnancy, and the great nutritive and nervous activity coincident with it, render the women more susceptible to such exciting causes as would manifest themselves in nervous outbreaks.

Because of the attitude of obstetric writers, the inexperienced may believe that these ailments will continue to the end of gestation and will cease not a moment earlier. This is also a popular

1. Read before the Buffalo Medical Club belief and, in consequence, many women fail to consult their physicians for serious, yes dangerous, conditions. Many women I have seen who have borne up during a weary nine months with troubles which could have been promptly relieved had they been given attention. Defective elimination of tissue waste poisons the nervous system. The maternal excretory organs in pregnancy must eliminate the combined waste of mother and child. Considering this and the more irritable state of the nervous apparatus in pregnancy, it is no wonder that slight defects in excretion make pronounced nervous effects.

About five years ago, I first came to realize that there are other causes for these than the mere existence of pregnancy.

The first case which taught me this was an exaggerated one.

She was a healthy appearing robust multipara, about twenty-five years of age; seven months in her second pregnancy. Her appetite was poor, tongue furred, bowels nearly regular, but she was nervous, despondent, and sleepless. For nearly a month she had slept only a small part of the night. There was no anasarca; the amount of urine excreted was about normal, and contained no albumen or casts. The skin was harsh and dry. She had not perspired perceptibly for the past three months. After watching her for a week or more, in the meantime giving her anti-spasmodics and hypnotics without much relief, I again examined her urine, and found the quantity sufficient but the specific gravity was low. As soon as I began to stimulate the elimination of solids by saline diuretics, her general condition improved, her nervousness decreased, and she could again sleep during a large portion of the night.

To complete the result, I stimulated her skin to activity by the hot steam bath, the result of the combined treatment being a complete restoration to a state of mental and nervous quietude.

In the meantime, I have seen several more cases in hospital and private practice, presenting a wide variety of nervous phenomena, most of which could be traced to defective excretion as the cause. The last half of pregnancy is the period in which it most frequently occurs. The usual conditions found are loss of appetite, a furred tongue, either constipation, a dry inactive skin, deficient quantity of urine—or a urine deficient in solids. Some or all of these may coexist. The usual train of nervous disturbances is nausea, pyrosis, backache or headache, neuralgia, general nervousness, or despondency and insomnia.

A pregnant woman should be more closely watched during pregnancy than is the custom. Her last three months, at least, should be passed under the direction of her expected attendant. His services during this period may never be absolutely necessary in any individual case. But, if aught arises, it will be known, and harm thereby prevented.

The urine should be examined frequently. By examination I do not mean that it be tested only for albumen. The entire quantity for twenty-four hours should be saved and measured once a week, at least, and a memorandum of the quantity and a sample of the same sent to the physician's office. While still fresh, it should be examined thoroughly enough to allow no possibility of abnormal conditions to escape him. A quantitative estimation of urea should be made. It is so easily done that I am in the habit of doing it at each examination. The condition of the patient's skin demands attention, and frequent recourse had to the warm vapor or hot-air bath, if it shows a sluggish tendency. The alvine excretions also demand constant supervision. The best hygienic surroundings, sufficient exercise in the sunlight, good nutritious food, tonics, and helps to better digestion and assimilation if needed, a care to the waste gates of the body, and if the woman begin her pregnancy with healthy organs and a normal vigor, she must come through her gestation and accouchment with fewer ailments, and with less drain upon her vitality, than by any other means.

In the treatment of defective elimination, I have found that best results can be had, in most cases, by a strict milk diet for a few days, laxatives, hot air, steam baths, or hot packs, and saline diuretics. Basham's Mixture, or the Liq. Ferri et Ammonii Acetatis, of the Pharmacopeia, I have found to be one of the best diuretics. In two cases, in which I suspected pressure upon the ureters by the lower uterine segment and contents a cause of the deficient urine, I placed the patient in a lateral prone position while sleeping, thus allowing the uterus and contents to be lifted out of the pelvis and decrease the pressure.

Nervous sedatives and hypnotics may be of use in the treatment, but they do not reach the cause. Little benefit comes from their use while the cause of the trouble exists.

64 Richmond Avenue.

---

A Koch laboratory is to be erected at the World's Fair, this Summer, to illustrate in a thoroughly practical manner, the eminent professor's work in microbic culture.

